# Editorial: Implementing mental health prevention and promotion programs: a sustainable approach - volume III

**DOI:** 10.3389/fpsyg.2026.2002851

**Published:** 2026-10-12

**Authors:** Agustín Albesa, Santiago Gascón-Santos, Yago Pérez-Montesinos, Mireya Zamora-Macorra

**Affiliations:** 1 Department of Psychology and Sociology, University of Zaragoza, Zaragoza, Spain; 2 Department of Health Care, Universidad Autonoma Metropolitana, Mexico City, Mexico

**Keywords:** mental health prevention, mental health promotion, public health, pyschological wellbeing, resilience, school mental health

Mental health promotion has become one of the guiding principles of public health, understood as an active investment in the conditions of all kinds that enable people to be healthy and happy. The third volume of this series on sustainable mental health prevention and promotion programs brings together a variety of contributions from diverse latitudes around the world and across broad generational groups, offering evidence of the relevance of the living conditions that impact mental health, ranging from improving communication between parents and children to the organizational climate within police forces, from the experience of a daycare center in western China to the effectiveness of a helpline for people in desperate situations that has received more than two million calls in Spain. This Research Topic received 45 manuscripts between October 2025 and August 2026, of which 19 were accepted; these contributions are diverse both in terms of methodology and content.

From a general perspective, Hernández-Paez et al. analyzed a synthesis of the state of the art based on 386,671 publications on mental health spanning more than a century. Scientific output varies across different fields and continents. The Americas and Europe account for more than three-quarters of the publications, but show a weak or non-significant association with disability-adjusted life years and suicide mortality, whereas Africa and Southeast Asia exhibit research more closely linked to conditions that promote mental health.

At the care level, Hernández-Díaz et al. evaluated two decades of records from the “*Teléfono de la Esperanza*” (a helpline for desperate situations) in Spain, concluding that loneliness and communication difficulties, and not just clinical symptoms, are the most frequent reasons for contacting this service, with a marked increase with age, reaching 77% of calls among people over 75 years old.

Many authors point to early childhood as the critical period during which these communication problems develop. Feng et al., based on a study of more than 20,000 mother/father-child pairs in Chinese nursery schools, found that parental verbal responsiveness was associated with fewer difficulties and better social behavior in pre-schoolers, and that these benefits could extend into adolescence.

Specifically, in an effort to explore the mechanisms that may help prevent depression during adolescence, Huang et al. investigated how different family communication patterns might be related to depressive symptoms. These authors found that greater development of hedonic capacity and lower expression of anger may play a key role in the relationship between family communication patterns and depression in adolescence, which has important implications for intervention and mental health promotion. On the other hand, Li X. et al. showed that the perception of parental rejection predicted depression in adolescents, while Li Y. et al., through a study of social networks, found that childhood adversity is linked to social anxiety in college students. From the perspective of mental health promotion, in addition to improvements in communication channels, researchers have examined the benefits of developing a curriculum that integrates family members' participation in physical activities into these programs (Schwartz et al.).

Continuing with studies on adolescents and young adults, Guo et al., identified three mental health profiles distinct from the traditional two-factor model—a “complete mental health” profile (61.30%), one “with difficulties” (31.51%), and a third, minority, and more severe “with greater difficulties” profile (7.19%)—in which cognitive reappraisal acts as a protective factor and expressive suppression as a risk factor across all profiles. Therefore, in seeking to implement health promotion programs for young people, it has been found that school psychologists are truly effective when their work is carried out as a daily and ongoing activity, rather than as an *ad hoc* emergency resource (Iudici et al.). Along these lines, Margaretha et al., in analyzing 161 schools in Indonesia—public, private, and religious—observed that public schools implement mental health promotion programs more successfully than the other two sectors, and that differences in participation across school types correlated with the programs' effectiveness.

Turning to the field of higher education and the potential benefits of digital tools, Hassan et al., based on a sample of 384 medical and health sciences students in Saudi Arabia, found a high prevalence of anxiety, depression, and stress, along with persistent barriers to access—primarily stigma and cost—that prevent students from seeking help even where such services exist. Along these lines, Xing et al., applying an extended model of technology acceptance to 327 college students, found that facilitating conditions, social influence, and perceived enjoyment together explained 74% of the variance in behavioral intention to use virtual reality as a mental health support—a remarkable figure for an emerging technology; while Laane et al. explored a different technological approach and demonstrated, in a sample of 370 matched adults (from the general population and those with mental disorders) aged 18 to 87, that 6 months of online brain training reduced psychological distress and improved resilience and quality of life equally in both groups, although cognitive improvements were limited to the general population.

In the occupational field, Berlanga Silvente et al. synthesize recent literature on mental health among police officers into an Integrated Model built on five protective factors—individual and collective resilience, organizational support, emotional literacy, professional identity, and psychosocial climate—a framework that discourages the mere pursuit of professional wellbeing through isolated interventions and emphasizes the need to ensure that organizational design supports health promotion.

Beyond mental health in work and educational settings, some of the articles focus on variables of everyday life. Yu et al., by cross-referencing the 2021 China General Social Survey with provincial ecological data, found that both the perceived and objective quality of “blue spaces”—bodies of water, their quality, and their sufficiency—predict happiness, expanding the hypothesis that humans' innate tendency or affinity to seek connections with nature goes far beyond green spaces and highlighting water resource management as a public health tool. Nosè et al. provide complementary evidence from a very different setting: a structured tour of a museum in Verona improved psychological distress and symptoms of depression and anxiety among participants, suggesting that cultural institutions are also valuable allies in health promotion—a role that remains underutilized. For their part, Lutz et al. combined the use of wearable sensors with repeated daily questionnaires among 53 adolescents to show that the number of steps taken and time spent standing were positively associated with momentary mood, while sedentary time and episodes were negatively associated with mood.

Morillo-Sarto et al. modeled the suicidal process in 918 Spanish adolescents, identifying two dominant pathways from psychological distress to suicide attempts: one pathway follows the entire symptomatic sequence typical of this process, while another, more direct pathway traces the progression from thoughts of hopelessness and the desire to be dead to the attempt itself; they argue that prevention efforts should also target these less intense ideations, not just explicit suicidal ideation. As a complement to this clinical approach, Villarreal-Otálora et al. validate the English and Spanish versions of a scale that measures the self-efficacy of Latino parents in communicating suicide prevention with their adolescent children, in a sample of 634 parents (220 born abroad and 414 born in the United States), and they find distinct factor structures and reliability patterns between the two groups, which supports the creation and use of culturally adapted—rather than merely translated—prevention materials.

Taken together, these 19 studies map mental health from infancy to old age and from the mother-child dyad to the organizational design of a police corporation. These studies—while not an exhaustive compilation of current research in this field—all emphasize the fact that the sustainable promotion of mental health is not based on a single-pronged intervention, but rather involves a family of context-specific practices—parental communication, school policy, recreational programming, feedback from mobile devices, helpline infrastructure, organizational design—united by a commitment to go beyond diagnosis and crisis. The common thread among these very different scenarios is not that they constitute a single mechanism, but rather that they form part of a shared discipline that rigorously measures outcomes, addresses the groups or segments left out by each intervention, and builds structures and processes capable of enduring once the initial funding for a specific project has run out.

